# Innate immune surveillance of the circulation: A review on the removal of circulating virions from the bloodstream

**DOI:** 10.1371/journal.ppat.1010474

**Published:** 2022-05-05

**Authors:** Stephanie E. Ander, Frances S. Li, Kathryn S. Carpentier, Thomas E. Morrison

**Affiliations:** 1 Department of Immunology and Microbiology, University of Colorado School of Medicine, Aurora, Colorado, United States of America; 2 Department of Natural Sciences, Greensboro College, Greensboro, North Carolina, United States of America; University of North Carolina at Chapel Hill School of Medicine, UNITED STATES

## Abstract

Many viruses utilize the lymphohematogenous route for dissemination; however, they may not freely use this highway unchecked. The reticuloendothelial system (RES) is an innate defense system that surveys circulating blood, recognizing and capturing viral particles. Examination of the literature shows that the bulk of viral clearance is mediated by the liver; however, the precise mechanism(s) mediating viral vascular clearance vary between viruses and, in many cases, remains poorly defined. Herein, we summarize what is known regarding the recognition and capture of virions from the circulation prior to the generation of a specific antibody response. We also discuss the consequences of viral capture on viral pathogenesis and the fate of the captor cell. Finally, this understudied topic has implications beyond viral pathogenesis, including effects on arbovirus ecology and the application of virus-vectored gene therapies.

## Author summary

Limiting the amount of virus freely circulating in the bloodstream can be important for controlling viral pathogenesis and transmission. However, despite early advances, this field of study has become overlooked and understudied. Innate immune cells in the liver and spleen constantly survey and remove from circulation viral particles without the aid of virus-specific antibody. The details of these host–virus interactions, and the consequences thereof, remain unknown for many viruses. Yet, understanding this phenomenon has implications not only on bettering our understanding of disease progress, but also on arbovirus ecology and the development of effective virus-vectored gene therapies.

## Introduction

It has been established for over a century that foreign particles introduced intravenously (i.v.) into vertebrates are rapidly removed from circulation [1]. In 1904, Ribbert reported lithium carmine solution injected i.v. “vitally stained” a specific subset of cells [1]. Through careful analysis of tissues following i.v. dye inoculation, Aschoff defined cells able to sequester vital dyes from the blood as the “reticuloendothelial system” (RES) [1]. Today, the RES is understood to be composed of macrophages, circulating monocytes, and endothelial cells that remove from circulation particulates like cellular debris, immune complexes, and microbes. The role of specific populations, cell surface receptors, and humoral components in microbial vascular clearance can be elucidated using drugs for selective cellular depletion [2], genetic and conditional knockouts (KO) in mouse models [3,4], and advanced live imaging techniques [5,6].

Since initial studies in the late 19th century, there have been great leaps in our understanding of microbial vascular clearance. However, most mechanistic studies focus on clearance of blood-borne bacteria, and limited mechanistic reports exist on viral vascular clearance. Herein, we introduce key cell types of the liver and spleen demonstrated to mediate the bulk capture of circulating virions prior to generation of a specific antibody response and summarize known host and viral mechanisms orchestrating clearance of specific viruses from mammalian circulation. We also discuss consequences of vascular clearance on viral pathogenesis and additional implications of these studies on both arbovirus ecology and virus-vectored gene therapies.

## The blood-filtering organs

Beginning in the late 1950s, the importance of circulating blood in promoting viral dissemination garnered scientific interest in the role of host innate immune defenses against viremia. One of the first papers to describe the RES as an innate defense against circulating virions was published in 1959 using ectromelia virus (ECTV; mousepox) [7]. Applying techniques previously developed to study vascular clearance of inert particles, Mims found i.v. inoculated ECTV was rapidly removed from circulation and colocalized with cells lining the liver sinusoids, likely Kupffer cells (KCs) or liver sinusoidal endothelial cells (LSECs) [7] (see Poxviruses section). Since this initial study, multiple and diverse viruses have been examined. In general, while clearance rates vary, virion removal is often rapid and mediated predominantly by the liver, although there is also evidence of spleen involvement (**Table 1**). As an aside, it should be noted that these studies on viral capture from the bloodstream assume vascular dissemination occurs via free viral particles. However, hematogenous spread of some viruses, such as cytomegalovirus, primarily occurs in a cell-associated manner—which adds another layer of complexity [8].

**Table 1:** Host mechanisms of viral clearance.

Organ	Cell type	Host mediator	Virus
Liver	KCs	Natural antibodies	Gene therapy vector: AdV [9–11]
		CRIg	Gene therapy vector: AdV [12]
		Complement	Gene therapy vector: AdV [10,12]
		SRs	Gene therapy vector: AdV [10,13,14]
		SR-A1 (MSR1)	Gene therapy vector: AdV [15,16]
		SR-A6 (MARCO)	Arbovirus: CHIKV, RRV, and ONNV [17]
		SR-F1 (SREC-I)	Gene therapy vector: AdV [15,16]
		Platelets	Gene therapy vector: AdV [18]
		GAGs	Gene therapy vector: AAV [19]
		ND	Blood-borne virus: HIV [20] Arbovirus: SFV [21], small-plaque variants of VEEV [22], and VSV [23] Gene therapy vector: AdV [24,25] Other: CPXV [26], DHBV [27], ECTV [28], LCMV [29], NDV [30], BKPyV [31], JCPyV [31], and RABV [32]
	LSECs	SR-A1 (MSR1)	Gene therapy vector: AdV [15,16]
		SR-F1 (SREC-I)	Gene therapy vector: AdV [15,16]
		GAGs	Gene therapy vector: AAV [19,33]
		ND	Blood-borne virus: HIV [20] Other: DHBV [27, 34], BKPyV [31], and JCPyV [31]
	Hepatocytes	Coagulation factors	Gene therapy vector: AdV [10,33,35–37]
	ND	Natural antibodies	Gene therapy vector: AdV [38]
		Complement	Gene therapy vector: AdV [38]
		SRs	Gene therapy vector: MV [39]
		GAGs	Arbovirus: MVEV [40], SINV [41], and VEEV [42]
		ND	Blood-borne virus: SIV [43, 44] Arbovirus: LGTV [45], MVEV [7], RVFV [46], VSV [30], and YFV [47] Gene therapy vector: AdV [48] Other: DHBV [34], ECTV [7,49], IFV [7,50], LCMV [29], and PV [7], RV [51]
Spleen	Marginal zone, MZMs, and MMMs	ND	Arbovirus: VSV [23,52,53] Gene therapy vector: AdV [48,54–57] Other: DHBV [27], BKPyV [31], JCPyV [31], HSV [58], and RABV [32,59]
	Red pulp and red pulp macrophages	ND	Arbovirus: VSV [23] Gene therapy vector: AdV [48,54] Other: BKPyV [31] and RABV [32,59]
	Macrophages	ND	Other: LCMV [29]
	ND	Natural antibodies	Arbovirus: VSV [60] Other: LCMV [60] and VACV [60]
		GAGs	Arbovirus: VEEV [42]
		ND	Blood-borne virus: SIV [43] Arbovirus: LGTV [45] and YFV [47] Gene therapy vector: AdV [48] Other: ECTV [49] and RV [51]
Kidney	Endothelial cells	ND	Other: BKPyV [31] and JCPyV [31]
	ND	ND	Other: LCMV [60]
Lung	ND	ND	Blood-borne virus: SIV [43] Arbovirus: LGTV [45] Other: RV [51,61]
Lymph node	ND	ND	Blood-borne virus: SIV [43]
ND	Macrophages	ND	Arbovirus: YFV [62] Other: JUNV [63] and VACV [64]
	Platelets	Glycophorin A	Other: HAV [65]
	ND	Complement	Arbovirus: SINV [66] and WNV [67] Gene therapy vector: AdV [68]
		MBL	Arbovirus: DENV [67] and WNV [67]
		SR	Gene therapy vector: AAV [69]
		GAGs	Arbovirus: JEV [40], EMCV [70], SINV [71], VEEV [42,72], and WEEV [73]

AAV, adeno-associated virus; AdV, adenovirus; BKPyV, BK polyoma virus; CHIKV, chikungunya virus; CPXV, cowpox virus; DENV, dengue virus; DHBV, duck hepatitis B virus; ECTV, ectromelia virus/mousepox; EMCV, encephalomyocarditis virus; GAG, glycosaminoglycan; HAV, hepatitis A virus; HIV, human immunodeficiency virus; HSV, herpes simplex virus; IFV, influenza virus; JCPyV, JC polyoma virus; JEV, Japanese encephalitis virus; JUNV, Junin virus; KC, Kupffer cell; LCMV, lymphocytic choriomeningitis virus; LGTV, Langat virus; LSEC, liver sinusoidal endothelial cell; MBL, mannose-binding lectin; MMM, marginal zone metallophilic macrophage; MV, measles virus; MVEV, Murray Valley encephalitis virus; MZM, marginal zone macrophage; ND, not determined; NDV, Newcastle disease virus; ONNV, o’nyong’nyong virus; PV, poliovirus; RABV, rabies virus; RRV, Ross River virus; RV, reovirus; RVFV, Rift Valley fever virus; SFV, Semliki Forest virus; SINV, Sindbis virus; sIV, Simian immunodeficiency virus; SR, scavenger receptor; VACV, vaccinia virus; VEEV, Venezuelan equine encephalitis virus; VSV, vesicular stomatitis virus; WEEV, Western equine encephalitis virus; WNV, West Nile virus; YFV, yellow fever virus.

### Liver

**Fig 1:**
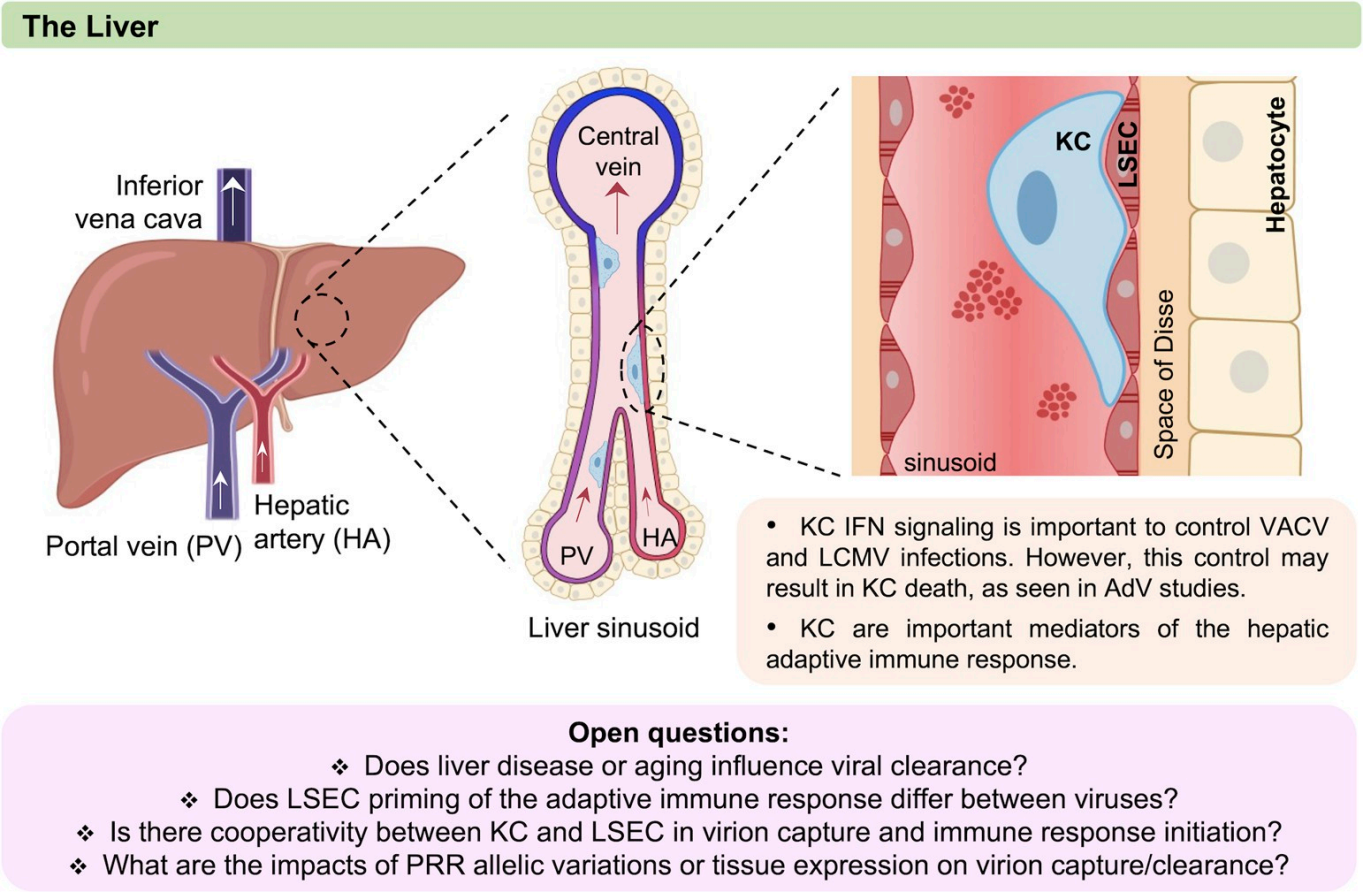
The liver sinusoid. There are 2 key cell types located in the liver sinusoid that have been shown to contribute to viral vascular clearance. Although in vitro studies suggest that LSECs, which form the liver endothelium, interact with certain viruses (e.g., AdV), KCs, which are the liver’s main tissue-resident macrophages, are responsible for clearing diverse circulating viruses (e.g., CHIKV and AdV) in vivo. In addition, KCs are important in controlling pathogenesis of viruses like LCMV. This figure was created with BioRender.com. AdV, adenovirus; CHIKV, chikungunya virus; HA, hepatic artery; IFN, interferon; KC, Kupffer cell; LCMV, lymphocytic choriomeningitis virus; LSEC, liver sinusoidal endothelial cell; PRR, pathogen recognition receptor; PV, portal vein; VACV, vaccinia virus.

The liver plays a critical role in immune surveillance and has evolved a number of features that promote efficient removal of foreign or unwanted molecules from the blood. Every minute, 1,500 mL flows through the human liver [74]. Blood is supplied from both the hepatic artery and the portal vein, exposing the liver to systemic and gut-derived microbes. In the liver, blood percolates through the honeycomb-like structure of the liver sinusoids [6,75]. Within the narrow sinusoids (5 to 10 μm in diameter in rodents [76–78]), blood flow rate is reduced [79], maximizing contact between blood contents and liver cells to allow recognition and removal of unwanted particles [75]. Lining the sinusoids are LSECs, which form a selective barrier between blood and hepatocytes. Attached to the luminal surface of LSECs are KCs, the tissue-resident macrophage of the liver (**Fig 1**). Both LSECs and KCs express a diverse array of pathogen recognition receptors (PRRs) at their surface to detect and remove unwanted pathogens from circulation (**Table 2**).

**Table 2:** Documented surface-expressed pattern recognition receptors of LSECs and KCs.

**LSECs (approximately 50% of nonparenchymal cells in liver) [80–88]**		
	***Mus musculus***	***Homo sapiens***
**SR**	SR-A1 (MSR1), SR-B1 (SCARB1), SR-B1.1 (SCARB2), SR-B2 (CD36), SR-E1 (OLR1), SR-E3 (CD206), SR-F1 (SREC-I), SR-G (CXCL16), SR-H1 (STAB1), and SR-H2 (STAB2)	SR-A1 (MSR1), SR-E1 (OLR1), SR-E3 (CD206), SR-F1 (SREC-I), SR-H1 (STAB1), and SR-H2 (STAB2)
**C-type lectins receptor**	Mannose receptor (CD206/SR-E3), LSECTIN (CLEC4G), DNGR-1 (CLEC9A), and L-SIGN (CLEC4M)	Mannose receptor (CD206/SR-E3), LSECTIN (CLEC4G), and L-SIGN (CLEC4M)
**Toll-like receptor**	TLR1-2 and TLR4	TLR4
**Fc receptor**	FcγRIIB and FcγRn	FcγRIIB
**KCs (approximately 20% of nonparenchymal cells in liver) [80–82,84,85,88–93]**		
	***M*. *musculus***	***H*. *sapiens***
**SR**	SR-A1 (MSR1), SR-A6 (MARCO), SR-B1 (SCARB1), SR-B1.1 (SCARB2), SR-B2 (CD36), SR-E2 (CLEC7A), SR-D1 (CD68), SR-G (CXCL16), SR-H2 (STAB2), SR-I1 (CD163), and SR-L (LRP1)	SR-A1 (MSR1), SR-A6 (MARCO), SR-B1 (SCARB1), SR-B1.1 (SCARB2), SR-B2 (CD36), SR-E1 (OLR1), SR-E2 (CLEC7A), SR-E3 (CD206), SR-D1 (CD68), SR-G (CXCL16), SR-I1 (CD163), and SR-L (LRP1)
**C-type lectins receptor**	Mannose receptor (CD206/SR-E3), CLEC4F, CLEC7A (SR-E2), CLEC6A, DCIR (CLEC4A2), and LSECTIN (CLEC4G)	Mannose receptor (CD206/SR-E3), CLEC7A (SR-E2), DC-SIGN (CD209), LSECTIN (CLEC4G), and CLEC6A
**Toll-like receptor**	TLR1-2 and TLR4-6	TLR2 and TLR4
**Fc receptor**	FcγRI, FcεRII, FcγRIII, FcγRIV, and FcγRn	FcαRI, FcγRIIA, FcγRIIB, and FcγRIII
**Complement receptor**	CR3 (ITGAM), CRIg (VSIG4), C3aR, and C5aR	CR1 (CD35), CR3 (ITGAM), CR4 (ITGAX, ITGB2), CRIg (VSIG4), C3aR, and C5aR

KC, Kupffer cell; LSEC, liver sinusoidal endothelial cell; SR, scavenger receptor.

Unique to liver sinusoids, the liver endothelial lining is highly porous as it lacks a basement membrane, and LSECs are highly fenestrated [94–96]. The fenestrae (50 to 150 nm in diameter) are generally grouped together to form sieve plates that limit access of blood-borne particulates to the space of Disse and the underlying hepatocytes. LSECs have high clathrin-mediated endocytic capacity. Most often associated with pinocytosis of particles smaller than 200 nm [96–101], LSECs also are capable of phagocytosing larger latex beads following impairment of KC function [102].

KCs, positioned within the sinusoidal lumen, constitute the body’s largest population of tissue-resident macrophages and have multiple processes that extend into different sinusoids, which increases their surveillance area [103]. KCs are a self-renewing population [104–106], although circulating monocytes are capable of renewing the KC niche following selective KC depletion [3,107–109]. Capture of circulating viruses and other pathogens by KCs is generally considered to be mediated by phagocytosis. In vitro, direct comparison of endocytic activities of KC to that of splenic and peritoneal macrophages showed KCs to outcompete uptake of dextran and *Escherichia coli*, and in vivo, KCs supersede even splenic macrophages in the removal of dextran from circulation [110]. KCs also interact with other innate immune cells to defend against pathogens. Specifically, KCs can serve as a docking site for neutrophils to eliminate the bacteria trapped at the KC extracellular surface [111,112].

### Spleen

The spleen is another major contributor to removal of microbes in the bloodstream, as demonstrated in a study comparing contributions of splenic mass and blood flow on the clearance of *Streptococcus pneumoniae* in a rabbit model [113]. In contrast to sham- or hemi-splenectomized rabbits, those that underwent procedures to reduce splenic blood flow exhibited impaired rates of bacterial clearance, and completely splenectomized animals were unable to reduce the bacterial burden in the bloodstream [113].

**Fig 2:**
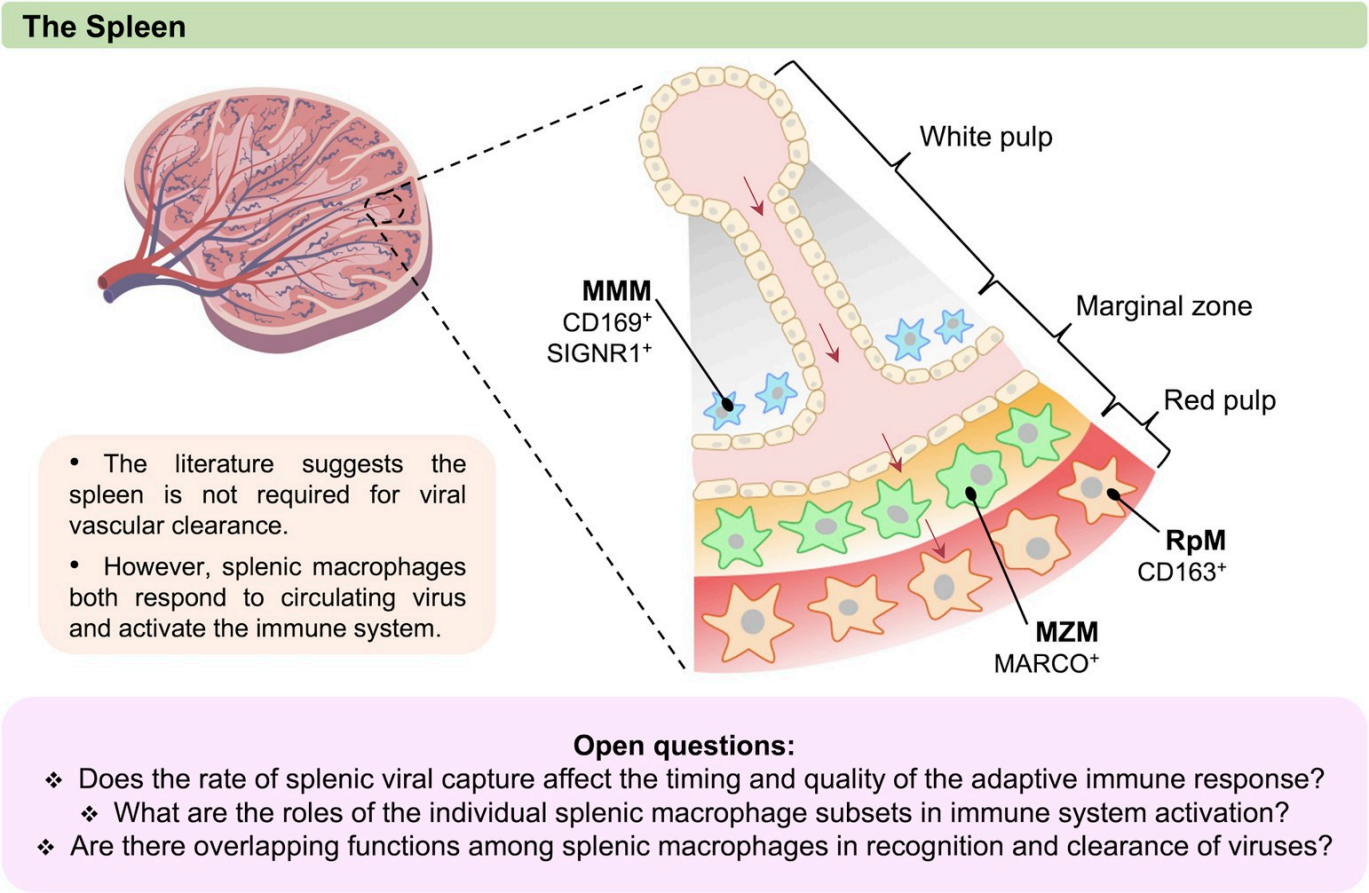
Macrophages of the spleen. Splenic macrophages also participate in the capture of circulating virus particles. There are 3 major splenic macrophage populations (MMM, MZM, and RpM), and they localize to distinct regions of the spleen. These macrophage subsets can be identified by their localization and the indicated key cellular markers. While the mechanisms by which specific splenic macrophage populations mediate viral clearance are not well understood, they are critical in activating immune responses to circulating viruses. This figure was created with BioRender.com. MMM, marginal zone metallophilic macrophage; MZM, marginal zone macrophage; RpM, red pulp macrophage.

In the spleen, macrophages mediate clearance of circulating particulates. The spleen has 3 major macrophage populations: red pulp macrophages, marginal zone macrophages (MZM), and marginal zone metallophilic macrophages (MMM). As arterial blood travels through the spleen, vessels passing through the white pulp open to form sinusoids within the marginal zone; blood then percolates through the marginal zone into the red pulp’s venous sinuses (**Fig 2**). MZM and MMM appear to be the main workhorses mediating clearance of blood-borne microbes [27,54,55,114,115], although red pulp macrophages also phagocytose bacteria [116,117] and deparasitize red blood cells of *Plasmodium* [118].

While there are examples of virus capture by splenic macrophages in the literature (such as adenovirus [AdV], discussed below), the spleen is typically dispensable or plays a minimal role in the clearance of virions from circulation. For example, splenectomized mice exhibit no defect in vascular clearance kinetics of chikungunya virus (CHIKV) [17], and examinations of viral biodistributions postclearance generally find the liver absorbs the bulk of the inoculum [5,7,17,25,30,31,42,51,119]. Yet, splenic capture of circulating microbes may have an important role in initializing an effective immune response necessary for the resolution of a natural infection [55,114].

### Blood-borne pathogens

Some of the most well-studied blood-borne viruses are human immunodeficiency virus (HIV), hepatitis B virus (HBV), and hepatitis C virus (HCV). For these viruses, viremia levels are indicators of disease progression during chronic viral infections, which is characterized by a viremia set point that is constant for years [120]. This constancy is likely due to continuous removal of viral particles from circulation to establish an equilibrium, as viral load would otherwise be expected to steadily increase over time [120]. The magnitude of this set point associates with disease progression [120–122]. For example, AIDS patients with high-viral set points tend to have a more rapid disease progression than those with low-viral set points [121,122].

Understanding host mechanisms mediating removal of these human-specific viruses from circulation is challenging. The most common method to estimate viral clearance rates uses antiviral therapy to halt virus production then measures plasma virion half-life [120]. Another technique is plasma apheresis, wherein plasma is removed from a patient and fluids returned at similar rates to maintain blood volume [120]. Viral plasma loads are compared before, during, and after apheresis. If the clearance rate due to apheresis is smaller than the calculated natural clearance rate, there will be little impact on plasma viral concentrations [120]. Using animal models, viral vascular clearance can also be examined following i.v. inoculation.

### HIV/SIV

In animal models, simian immunodeficiency virus (SIV) and HIV-1 viral particles are rapidly removed from circulation. Following i.v. inoculation into naive and SIV-infected rhesus macaques, newly inoculated SIV particles were quickly cleared from the plasma at an estimated half-life of 1.3 to 4.6 minutes [43,44]. Inoculated virus was not found in the blood’s cellular compartment, nor was it degraded when incubated in blood ex vivo, suggesting active removal of virions from circulation [43]. Another rhesus macaque study also identified rapid removal of HIV particles from circulation with half-lives of 13 to 26 minutes in naive animals [123]. A very small percentage of inoculated virus could be detected in the primate spleen, lungs, and lymph nodes [43,44]; however, the bulk of viral clearance from circulation was mediated by the liver [20,43,44]. In mice, inoculation of HIV-like particles resulted in clearance of 97% of the inoculum by 10 minutes [20], and HIV structural proteins env and gag were observed to associate with LSECs (approximately 88%) and KCs (approximately 12%) [20]. Studies in SIV macaque models suggest that captured virions are rapidly degraded, as only 30% of infused S35-labeled virus was detected at 1 hour postinoculation (hpi) [44], and no viral RNA was detected in tissues at later time points [43,123].

Human patient data also support a short half-life of circulating HIV particles, ranging from 28 minutes to no greater than 6 hours depending on methods used [124,125]. Correlating with animal data, HIV antigen and mRNA can also be detected in patient livers, particularly within KCs and to some degree within hepatocytes [126,127]. In vitro, both KC and LSEC primary cultures are capable of permissive HIV infection [128–130]. However, while liver samples of HIV-infected individuals (and SIV-infected macaques) have shown KCs stain positive for HIV antigen and nucleic acids, it remains uncertain whether KCs are able to support productive HIV/SIV replication in vivo [126,127,131–133]. In addition, host factors on KCs and LSECs responsible for mediating HIV-1 removal from circulation have yet to be identified.

### HBV

Initial estimates of HBV half-life in the circulation ranged from 1 to 3 days [134–138]. These estimates were calculated following antiviral treatment to arrest HBV replication. However, more recent studies have attempted to account for the delayed release of HBV virions assembled prior to the start of drug intervention. The first such study calculated a revised HBV half-life of 3.8 hours in a chimpanzee model [139]. A subsequent study comparing chronic disease patients categorized into low- and high-viremic groups estimated median half-lives of 2.5 minutes and 46 minutes, respectively [140], suggesting that clearance rates may be affected by viral load or underlying host factors. Interestingly, such distinction in clearance rates between low- and high-viremic patients was not observed in an immunodeficient mouse model (HBV half-life of 3 hours) [140]. It has been suggested liver hepatocyte expression of sodium taurocholate cotransporting polypeptide both mediates HBV removal from circulation and establishes liver infection [141].

### HCV

HCV is rapidly cleared from circulation with a half-life of a few hours in the blood, as calculated from antiviral therapy [142,143], plasma apheresis [124,144], and liver transplantation studies [145]. Liver transplantation studies indicate the liver is not only involved in HCV replication but also viral clearance from circulation, as immediately postprocedure liver transplant recipients exhibit significantly enhanced rates of viral clearance [145]. However, mechanisms mediating this clearance are unknown and could be due to infection of new hepatocytes, capture by reticuloendothelial cells of the donor liver, or a combination thereof.

### Arboviruses

Arboviruses are arthropod-borne viruses maintained in nature through transmission cycles involving hematophagous arthropod vectors and vertebrate hosts [146]. Viremia is an important determinant of arbovirus transmission efficiency, reservoir competency, and disease severity. Critical for arbovirus transmission, vertebrate hosts must produce a viremia of sufficiently high magnitude and duration to support infection of the arthropod vector from a blood meal. Beyond transmission, increased levels of viremia have also been shown to correlate with more severe disease outcomes for several arboviruses [147–152]. However, our understanding of arboviral viremia control is limited.

### CHIKV, RRV, and ONNV

**Fig 3:**
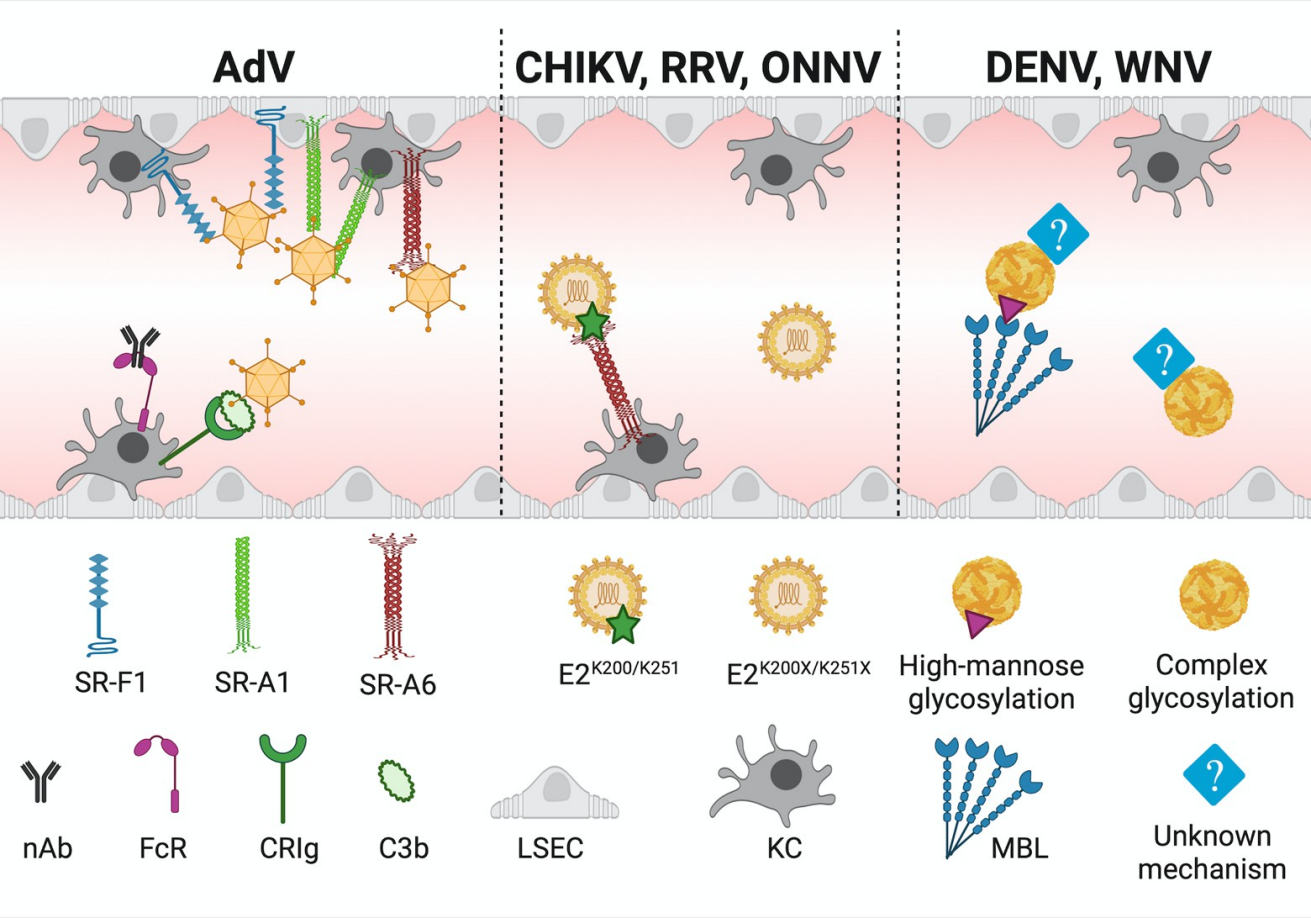
Mechanisms of viral capture. The liver appears to be the main mediator of viral vascular clearance. However, the specific mechanisms of removing virions from the circulation is distinct and virus-specific. The removal of AdV particles is mainly performed by KCs; however, some of the receptors shown to interact with AdV can also be expressed by LSECs (SR-F1 and SR-A1). In addition to SRs (SR-F1, SR-A1, and SR-A6), nAb, and CRIg also promote clearance of AdV from the bloodstream. For arthritogenic alphaviruses (CHIKV, RRV, and ONNV), clearance is mediated specifically by SR-A6 (MARCO) and KCs. However, particles that have a single point mutation to replace a lysine residue on the E2 glycoprotein (K200X for CHIKV and ONNV; K251X for RRV) evade capture. For the flaviviruses DENV and WNV, the type of virion glycosylation present affects clearance mediated by MBL. Specifically, MBL binds the high-mannose glycosylated virus particles, but not virions decorated with complex glycosylation. However, MBL is not the only mediator of DENV and WNV clearance, and it is clear another, as-yet-unknown mechanism also exists. This figure was created with BioRender.com. AdV, adenovirus; CHIKV, chikungunya virus; DENV, dengue virus; KC, Kupffer cell; LSEC, liver sinusoidal endothelial cell; MBL, mannose-binding lectin; nAb, natural antibodies; ONNV, o’nyong’nyong virus; RRV, Ross River virus; WNV, West Nile virus.

In mice, vascular clearance of arthritogenic alphaviruses CHIKV, Ross River virus (RRV), and o’nyong’nyong virus (ONNV) depends on the presence of scavenger receptor (SR) MARCO (SR-A6) (**Fig 3**) [17]. In wild-type (WT) mice, these viruses are efficiently cleared from circulation in less than 1 hour following i.v. inoculation [17]. Meanwhile, MARCO-deficient mice fail to remove i.v. inoculated virus, and following subcutaneous (s.c.) inoculation, they exhibit enhanced viral dissemination and worse disease outcomes [17].

MARCO-mediated clearance is specifically dependent on the presence of a particular lysine residue in the viral E2 glycoprotein. For CHIKV and ONNV, that critical lysine residue is at position E2-200 (K200), and for RRV, it is at position E2-251 (K251) [17]. Interestingly, substitution of any other residue, including another positive-charged residue, at these sites produces virions resistant to murine vascular clearance [17]. This lysine-specific vascular clearance phenotype suggests that the virion’s MARCO binding site may be sterically restrained. Alternatively, ONNV and CHIKV E2-K200 and RRV E2-K251 may be posttranslationally modified, as many possible lysine modifications exist [153], and SRs were first identified based on the capacity to recognize molecules such as low-density lipoprotein (LDL) with specific modifications on lysines including acetylation and oxidation [154].

### VEEV

Serum clearance studies with Venezuelan equine encephalitis virus (VEEV) have suggested correlations between virion–glycosaminoglycan (GAG) interactions and clearance from circulation. Specifically, VEEV strains that exhibit high affinity for GAGs in vitro are more swiftly removed from circulation than strains with reduced GAG-binding properties [42]. For example, mutations in VEEV that enhance virion–GAG interactions correlated with more rapid vascular clearance following i.v. inoculation of mice, with clearance mediated by the liver, spleen, lung, and kidney [42]. A similar finding was observed in a nonhuman primate model, wherein VEEV vaccine strain TC-83 (distinguished by a point mutation in the viral E2 glycoprotein that enhances GAG-binding in vitro [42,155]) was rapidly removed from circulation, while its virulent progenitor strain, TrD, was resistant [72]. Studies with other viruses also correlate the presence of virion GAG-binding mutations with rapid vascular clearance (alphaviruses: Sindbis virus (SINV) [41,71], Western equine encephalitis virus [73], Eastern equine encephalitis virus [156]; flaviviruses: Japanese encephalitis virus [40], Murray Valley encephalitis virus [40]; and picornavirus: Mengo virus [70]). Following clearance, these GAG-binding viruses are often liver localized [22,40,42,71], which is known to have high amounts of heparan sulfate (a class of GAGs) [157] and to mediate vascular clearance of heparan sulfate-binding proteins in vivo [158–161]. Given the GAG-binding properties of these viruses described above are also associated with attenuation in vivo [42,162–164], GAG-mediated vascular clearance is commonly hypothesized to control viremia and thus ultimately limit disease development [165].

### SINV

Studies with SINV have identified a role for host-specific posttranslational modifications in determining viral clearance kinetics from the serum. Specifically, the absence of sialic acid on the SINV virion is associated with enhanced complement C3 activation in vitro and complement-mediated enhancement of vascular clearance in vivo [166]. Comparison of mosquito- and mammalian cell–derived virus detected more sialic acid associated with the latter [166]; insect cells generally do not sialylate glycans, unlike mammalian cells [167]. Removal of sialic acid from mammalian cell–derived virus by neuraminidase treatment resulted in enhanced complement C3 activation in vitro, comparable to mosquito cell–derived virus [166].

### DENV and WNV

Investigations on vascular clearance of dengue (DENV) and West Nile (WNV) virus particles from circulation have implicated a role for mannose-binding lectin (MBL) (**Fig 3**). In vivo, MBL contributes to swift vascular clearance of DENV (<0.5 hpi), as MBL-A/C-deficient mice cleared DENV particles less efficiently compared with WT mice [67]. In vitro, murine MBL binds to DENV and WNV via terminal mannose N-linked glycans and activates the MBL-complement pathway to neutralize virus [67]. In addition, in vitro human MBL can neutralize all 4 DENV serotypes independent of complement [168]. Whether MBL-mediated activation of the complement pathway is necessary for its role in vascular clearance remains to be investigated. Moreover, the absence of MBL delayed, but did not abolish, the vascular clearance of DENV, suggesting that additional pathways also contribute to the clearance of DENV particles from murine circulation.

The ability of MBL to bind WNV is influenced by viral glycosylation [67]. MBL binds strongly to mosquito cell–derived WNV, but not mammalian cell–derived WNV [67]. This was associated with cell type–specific N-linked glycan chains [67], as mosquito cells produced viral particles with truncated, high-mannose N-linked glycan chains, while mammalian cells are capable of further processing these N-linked glycans into more complex chains [167]. Inhibiting formation of complex N-linked glycosylation during WNV propagation in mammalian cells yielded progeny virions with exposed high-mannose sugars. These virions were more susceptible to MBL deposition, and this effect of MBL recognition of WNV N-linked glycosylation was supported by in vivo vascular clearance experiments [67].

### VSV

The first report on the serum clearance of vesicular stomatitis virus (VSV) found it to be rapidly removed from circulation over a 5-minute period, and at 20 minutes pi, most of the infectious virus recovered was in the liver [30]—later shown to colocalize with KCs [23]. Although a more recent study analyzing VSV biodistribution at 2 hpi found more infectious virus in the spleen rather than the liver [60]; where virus colocalized with red pulp macrophages and MMMs [23,53]. Regarding splenic capture, VSV removal from circulation was heavily dependent on the presence of IgM natural antibodies, wherein splenic uptake was reduced by 2 to 3.5 logs at 1 hpi in antibody-deficient mice, but liver uptake was unaffected [60]. Furthermore, reconstitution of μMT mice (deficient in functional B cells and thus also natural antibody) with a single dose of normal mouse serum 30 minutes prior to i.v. inoculation of a lethal dose of VSV permitted 75% to 80% survival by 60 dpi (0% survival of nonreconstituted mice by 10 dpi) [60].

### Gene therapy vectors

Gene therapy delivery by viral vectors is an attractive method due to viruses’ ability to evade immunosurveillance and deliver nucleic acids to specific cell types. Because viral vectors can be administrated i.v., clearance of these vectors can influence efficacy, side effects, and half-life of the gene therapy.

### AdV

The most extensively studied virus on the topic of viral vascular clearance is human AdV 5. For the purposes of this review, we highlight only those details of AdV vascular clearance that complement and generate a more comprehensive description of the virus–host interactions mediating removal of viral particles from circulation in general. For more information on AdV vascular clearance and the innate immune response, please see the detailed reviews by Allen and Byrnes [169] and Atasheva and colleagues [170].

Following i.v. inoculation, AdV is rapidly removed from circulation [171–173] and primarily distributed to the liver in both mice and nonhuman primates [9,48,171,172,174], although splenic macrophages in the marginal zone and red pulp have also been shown to be involved [48,56,57]. In mice, greater than 96% of circulating virus is cleared by the liver within 10 minutes post-i.v. inoculation [173]. An in vivo imaging study of near-infrared–labeled AdV particles revealed virus particles accumulated within the liver as soon as 11 seconds post-i.v. inoculation and saturation of the liver-localized signal occurred by 3 minutes postinoculation [5]. Within the liver, AdV specifically localizes to KCs [9,12,48,174–176]. However, there is also evidence of AdV uptake by LSECs [172], and mouse strain differences can affect whether the bulk of AdV uptake is performed by KCs or LSECs [11]. With regard to the latter, it is likely allelic differences play a role in determining which cell types mediate viral vascular clearance. Data in the same study implied mouse strain-dependent differences may also result in differential degrees of splenic involvement, wherein clearance in BALB/c mice is dominated by the spleen and C57BL/6, the liver [11].

It has been suggested that SRs expressed on KCs are responsible for capturing circulating AdV, specifically SR-A1 (MSR-1) [15,16,177], SR-F1 (SREC-I) [15,16], and SR-A6 (MARCO) (**Fig 3**) [178]. Supporting a role for SRs, pretreatment of mice with SR inhibitors (poly[I], poly[G], and/or dextran sulfate) reduced KC-AdV association by 80% to 90% and, subsequently, promoted greater liver transfection [10,14–16].

Natural antibodies and complement also promote uptake of AdV particles by KCs (**Fig 3**). RAG1 KO mice, which are unable to produce natural antibodies due to nonfunctional B cells, exhibit a 75% decrease in KC viral burden, but serum clearance can be partially rescued by preinjection of WT naive mouse serum [10]. Natural antibodies bind AdV in vitro [10,179], and several other studies offer supporting in vivo evidence in RAG KO mice, as their hepatocytes are more highly transduced upon AdV i.v. inoculation (implying poor uptake by KCs) [9,11,179]. As for complement, C3 is activated in vivo following i.v. inoculation of AdV [68]. In vitro studies with AdV found C3 and C4 directly bind virions [10] and inhibit viral replication postinternalization [180,181]. Furthermore, CRIg expression by KCs contributes to AdV vascular clearance. CRIg-deficient mice clear AdV from circulation less efficiently, and data suggest reduced uptake of viral particles by CRIg-deficient KCs [12]. Other hematogenous host factors can also promote AdV resistance to KC-capture. For example, binding of coagulation factors to AdV promotes hepatocyte transduction [10,33,35–37,175] and thus, by extension, escape from KC entrapment.

In addition to host determinants of clearance, several studies examined virion features affecting clearance and biodistribution of circulating AdV. A single-point mutation in the virion fiber protein (Y477A), known to ablate binding to the AdV entry receptor CAR (coxsackie and AdV receptor), delayed viral clearance from the bloodstream following i.v. inoculation [182]. Meanwhile, a different fiber mutation also known to disrupt CAR-binding (S408E-P409A) found no impact on viral capture by KCs [183]. These data suggest 2 mechanisms to remove AdV from circulation: KCs acting independent of CAR and a non-KC cell population dependent on CAR. Virion features that specifically affect KC uptake are the fiber and hexon proteins. Chimeric AdV with different serotype knob-domains of fiber (Ad35 and Ad9) resulted in varying degrees of KC association [184]. This is supported by in vitro data wherein pretreatment of primary KC with knob protein decreases AdV uptake [177]. Hexon protein also appears to mediate KC interactions, specifically through the hypervariable regions (HVRs). Chimeric Ad5 expressing the HVR of Ad6 results in 10-fold enhanced hepatocyte transduction and reduced KC loss (implying better KC evasion; see KC response section) [185]. Similarly, modification of the hexon HVR to enhance virion PEGylation caused 10- to 40-fold enhancement of hepatocyte transduction [13]. This enhancement is thought to be due to KC evasion, as pretreating mice to deplete KC did not produce any additive effects [13].

### MV vector

Clearance of measles virus (MV)-like particles from murine circulation is rapid, with a half-life of 1 minute and undetectable plasma virus levels by 30 minutes pi [39]. These clearance kinetics were measured in the absence of natural antibodies using severe combined immunodeficiency (SCID) mice, and clearance of MV-like particles appears to be primarily mediated by CD68^+^ macrophages of the liver and spleen. Because pretreatment with SR inhibitors (poly[I], poly[G], and dextran sulfate) reduced, but did not eliminate, viral uptake by the liver and spleen, a SR seems to be partially responsible for MV clearance. However, it is evident a second, poly[I]-insensitive mechanism of clearance exists [39].

### Poxviruses

A series of studies by Mims in 1959 analyzed the serum clearance of mousepox virus [7,49]. By 2 to 3 minutes post-i.v. inoculation, 90% of the inoculated mousepox virus was removed from circulation, and analysis of virus burdens in the tissues at 5 minutes pi found 95% of the inoculated virus was present in the liver, while the spleen accounted for only 4% [7]. The amount of infectious virus detected in the liver declined over time, suggesting viral particles were destroyed. Microscopic examination identified virus was captured by liver littoral cells (KCs and LSECs) but not hepatocytes [7]. Despite the rapid viral clearance from circulation, Mims noted a small fraction of inoculated virus persisted in the bloodstream. This residual virus associated with platelets, and when reinoculated into a naive mouse, remained relatively resistant to vascular clearance [7].

Building on this earlier work, a 2017 study on dissemination of vaccinia virus (VACV) found i.v. inoculation of low viral doses (100 and 1,000 plaque-forming units [PFU]) unable to effectively disseminate to murine ovaries [64]. However, depletion of phagocytic cells via clodronate permitted viral dissemination [64], suggesting that macrophages mediate the capture of VACV from circulation. In contrast, depletion of dendritic cells (DCs) in CD11c-DTR transgenic mice did not alter VACV dissemination [64]. In congruence, an earlier observation described that pretreatment of mice with thorotrast (which impedes phagocytic activity) also inhibited VACV vascular clearance [21]. However, the fate of VACV following vascular clearance appears to be tissue dependent. Hepatic capture results in viral destruction, as VACV antigen was only detected at early time points post-i.v. inoculation and became undetectable after 1 hpi [21]. In contrast, splenic uptake of VACV by MZMs [64] and MMMs [186] results in productive infection.

Natural antibodies have been proposed to promote splenic uptake of VACV, as μMT mice (deficient in functional B cells) exhibited both decreased serum clearance of the virus and decreased titer of virus in the spleen [60]. However, the liver may compensate for decreased splenic uptake as absence of natural antibodies was also associated with a modest increase in liver viral titer [60].

### Fate of viral capture

In general, rapid viral vascular clearance is associated with reduced viral pathogenesis, as seen in animal studies specifically impairing or depleting RES phagocytes via pharmaceuticals (e.g., thorotrast and clodronate-loaded liposomes). For example, s.c. inoculation of Semliki Forest virus (mimicking the natural route of inoculation for this arbovirus) into thorotrast-treated mice produced accelerated and heightened viremia compared with untreated controls [21]. Similarly, RES impairment enhanced herpes simplex virus 2 mortality [187] and promoted LCMV replication and viremia development [29] in murine models. Interestingly, while clodronate-treated, LCMV-infected mice were able to mount an initial virus-specific cytotoxic T lymphocyte (CTL) response, these T cells soon exhibited an exhausted T-cell phenotype as measured by an in vitro killing assay [188]. Similarly, a study of LCMV infection in *op/op* mice (that naturally lack MZMs but retain KCs [189,190]) also found disease development associated with exhausted CTLs or an immunopathologic CTL response [53].

Another method to investigate the impact of vascular clearance on disease severity is the utilization of specific viral mutants with altered clearance kinetics. One such mutation in the capsid of the hepatitis A virus (HAV) promoted faster serum clearance than WT virus due to its stronger affinity for glycophorin A expressed on erythrocytes [65]. Competition experiments, where differing amounts of WT and mutant HAV were co-inoculated i.v., showed that the mutant was specifically removed from circulation at a faster rate than WT virus [65]. This more rapid clearance correlated with less productive liver infection [65]. Similarly, a single-point mutation in the E2 glycoprotein of CHIKV, RRV, and ONNV made virions completely resistance to vascular clearance [17]. Following s.c. inoculation, this point mutation enhanced CHIKV dissemination, viremia, and subsequent disease severity [17,191]. From these studies, it is evident the RES is an important modulator of viral pathogenesis.

### KC and liver-mediated T-cell response

Following uptake of circulating viral particles, KCs restrict viral gene expression and replication in a manner dependent on signaling through the type-I interferon receptor (IFNAR). Upon VACV vascular clearance, viral replication was controlled by KC IFNAR signaling and promoted host survival [192]. Despite lack of detectable type I interferon (IFN-I) in the serum [193], a local, hepatic IFN-I response controlled viral replication [192]. Likewise, KC IFNAR signaling controlled LCMV infection [29] and was associated with a rapid influx of inflammatory monocytes to the liver [194]. In vitro, KCs isolated from human liver specimens phagocytize and degrade purified DENV-1 particles [195], producing antiviral cytokines, including IFN-⍺, interleukin (IL)-6, and tumor necrosis factor alpha (TNF⍺), in response to DENV-1 uptake.

However, KC–virus interactions may also result in KC death. For example, while most AdV particles captured by the liver are degraded [173,196], as evidenced by poor or failed transduction of KCs and LSECs [9,197,198], this control of viral infection also comes at a cost for the KC. Membrane disruption by the AdV capsid protein decreases the KC population size [25,183,199]. Consequently, this mutual destruction of KC and AdV could provide a window of opportunity for a secondary infection to disseminate unchecked until KC compartment repopulation.

Capture of circulating virions by the liver can affect development of an antiviral T-cell response (for a review on liver immunosurveillance, please see [200]). LSECs are suggested to activate T cells against circulating antigen; however, their priming of naive T cells typically produces tolerant or regulatory T-cell responses [200 201]. While KCs are generally skewed to promote a tolerogenic T-cell response [202], they can also induce an antiviral T-cell response analogous to that observed in the secondary lymphoid organs [203,204]. Specifically, KC-targeted uptake of virus has been shown to produce robust, effective CTL responses [203,205], even in the absence of hepatic DCs [203].

### Splenic macrophage response

While splenic macrophages do not appear to play a dominant role in the physical removal of circulating virions (as there are no reports of splenectomized animals failing to clear virus from circulation), their participation does impact the immunological responses to infection. For example, murine MZMs and MMMs are potent producers of IFN-I in response to i.v. inoculation of UV-inactivated herpes simplex virus 1 [58,115], while no IFN-producing cells were detected in the liver [58]. Likewise, AdV capture by marginal zone, MARCO^+^ macrophages elicited an inflammatory response [55]. Specifically, these MZMs recruited neutrophils to the spleen marginal zone leading to destruction of virus-associated macrophages [55].

Meanwhile, several studies with AdV [56,57] and VSV [23,52] have demonstrated a role for MMMs in promoting the development of strong B and T-cell responses. During infection, MMMs capture circulating virions but, unlike KCs and other splenic macrophage subpopulations, permit viral replication as a means of amplifying viral antigen for delivery to DC. Viral replication is supported by both the nonresponsiveness of MMMs to IFN-I due to expression of ubiquitin-specific peptidase 18 (USP18, a negative regulator of IFNAR signaling) [23] as well as the effects of TNF secreted by CD11b^+^/CD11c^−^/Ly6C^+^/Ly6G^+^ cells [52]. MMM-amplified viral antigen can then be cross-presented by DCs to prime CTLs [23,52,56, 57], eliciting a biased response to major histocompatibility complex (MHC)-I–binding peptides [57]. In the absence of DCs, an adaptive immune response is still elicited, albeit to a lower degree [57].

## Conclusions

The literature contains a vast variety of papers on the kinetics of viral vascular clearance and subsequent biodistribution. If susceptible to vascular clearance, most virions are rapidly cleared from circulation by the liver, with some splenic participation (**Table 1**). The mechanisms orchestrating clearance vary between viruses, and in some cases, the RES may utilize multiple, redundant avenues to capture a virion, as seen with DENV [67] and MV [39]. Interestingly, host mechanisms of viral vascular clearance may supersede viral interactions with receptors identified in vitro, as shown with AdV. For example, different AdV serotypes may use the same receptor in vitro, yet exhibit different biodistributions following i.v. inoculation [119].

While the literature describes a clear role for the RES-mediated removal of viral particles from circulation and importance in controlling viral pathogenesis, only a handful of studies have delved deeper to examine immunological responses elicited in specific RES cell populations following viral vascular clearance and the fate of captured virions [23,52,53,56,57,186,205].

Furthermore, it is unclear how aging or illness that disrupts integrity of the RES system (e.g., liver or spleen diseases) affects clearance of circulating viruses, although some studies observed impaired bacterial clearance in patients suffering liver cirrhosis [206, 207]. Elucidating virus–host interactions and downstream consequences (and in different physiological conditions) will enhance our understanding of the application of virus-vectored gene therapies, the impact of vascular clearance (or failure thereof) on viral pathogenesis and disease severity, and even the ecology of arthropod-borne viruses.
